# A cross-sectional survey to evaluate knowledge, attitudes and practices (KAP) regarding seasonal influenza vaccination among European travellers to resource-limited destinations

**DOI:** 10.1186/1471-2458-10-402

**Published:** 2010-07-07

**Authors:** Alena Pfeil, Margot Mütsch, Christoph Hatz, Thomas D Szucs

**Affiliations:** 1 Institute of Social- and Preventive Medicine, University of Zurich, Hirschengraben 84, 8001 Zurich, Switzerland; 2 Division of Epidemiology and Prevention of Communicable Diseases and World Health Organization (WHO) Collaborating Centre for Travellers' Health, Institute of Social- and Preventive Medicine, University of Zurich, Hirschengraben 84, 8001 Zurich, Switzerland; 3 Division of Medical Economics, Institute of Social- and Preventive Medicine, University of Zurich, Hirschengraben 84, 8001 Zurich, Switzerland

## Abstract

**Background:**

Influenza is one of the most common vaccine-preventable diseases in travellers. By performing two cross-sectional questionnaire surveys during winter 2009 and winter 2010 among European travellers to resource-limited destinations, we aimed to investigate knowledge, attitudes and practices (KAP) regarding seasonal influenza vaccination.

**Methods:**

Questionnaires were distributed in the waiting room to the visitors of the University of Zurich Centre for Travel' Health (CTH) in January and February 2009 and January 2010 prior to travel health counselling (CTH09 and CTH10). Questions included demographic data, travel-related characteristics and KAP regarding influenza vaccination. Data were analysed by using SPSS^® ^version 14.0 for Windows. Differences in proportions were compared using the Chi-square test and the significance level was set at p ≤ 0.05. Predictors for seasonal and pandemic influenza vaccination were determined by multiple logistic regression analyses.

**Results:**

With a response rate of 96.6%, 906 individuals were enrolled and 868 (92.5%) provided complete data. Seasonal influenza vaccination coverage was 13.7% (n = 119). Only 43 (14.2%) participants were vaccinated against pandemic influenza A/H1N1, mostly having received both vaccines simultaneously, the seasonal and pandemic one. Job-related purposes (44, 37%), age > 64 yrs (25, 21%) and recommendations of the family physician (27, 22.7%) were the most often reported reasons for being vaccinated. In the multiple logistic regression analyses of the pooled data increasing age (OR = 1.03, 95% CI 1.01 - 1.04), a business trip (OR = 0.39, 95% CI 0.17 - 0.92) and seasonal influenza vaccination in the previous winter seasons (OR = 12.91, 95% CI 8.09 - 20.58) were independent predictors for seasonal influenza vaccination in 2009 or 2010.

Influenza vaccination recommended by the family doctor (327, 37.7%), travel to regions with known high risk of influenza (305, 35.1%), and influenza vaccination required for job purposes (233, 26.8%) were most frequently mentioned to consider influenza vaccination.

**Conclusions:**

Risk perception and vaccination coverage concerning seasonal and pandemic influenza was very poor among travellers to resource-limited destinations when compared to traditional at-risk groups. Previous access to influenza vaccination substantially facilitated vaccinations in the subsequent year. Information strategies about influenza should be intensified and include health professionals, e.g. family physicians, travel medicine practitioners and business enterprises.

## Background

Pandemic and seasonal influenza are still a challenging field of the public health system. Influenza - a mild to severe respiratory infection caused by RNA viruses of the family Orthomyxoviridae - is one of the most common vaccine-preventable disease in travellers. Worldwide, between 250'000 and 500'000 deaths are estimated to be due to seasonal influenza infection each year [1]. Influenza is also responsible for tremendous economic costs both from admissions to hospital and loss of productivity [2]. Influenza affects all age groups and is usually self-limited. Common symptoms include acute fever, muscles pain, headache, cough and chills [3]. Special risk groups, such as very young children, the elderly and those suffering from chronic lung or heart diseases are at risk for serious influenza complications, e.g. bacterial pneumonia [4,5]. Influenza reaches peak prevalence in winter in the Northern hemisphere (Nov-Apr) - as well as in the Southern hemisphere (Apr-Oct) and circulates year-round in the tropics [6,7]. Seasonal influenza vaccination is an effective prevention strategy and is therefore routinely recommended for special risk groups [8,9]. Of note, the seasonal influenza vaccine recommendations of the U.S. Centres for Disease Control were recently expanded and include now about 80% of the population [10].

Influenza is known to be a quite frequent infection among travellers to tropical and subtropical destinations compared to other infections, e.g. vector-borne ones. About one of hundred travellers abroad gets infected [7]. The risk of infection depends on the travel destination and the season. Travellers crossing hemispheres may be confronted with different antigenic variants of the influenza virus. By returning home, the new variant may be transmitted to contact persons [11]. The first pandemic of the 21st century has highlighted the need for international influenza prevention strategies [12].

The objective of this study was to investigate the vaccination coverage as well as knowledge, attitudes and practices (KAP) regarding influenza vaccination among travellers to resource-limited countries to improve or adapt current preventive strategies.

## Methods

Two cross-sectional surveys were conducted at the University of Zurich Centre for Travel' Health during January and February 2009 and January 2010, respectively. Self-administered, anonymous questionnaires including 16 items were distributed to travellers waiting for pre-travel health advice. Participation was voluntary. Individuals above 17 years, understanding German or English, residing in Switzerland and planning to travel to a resource-limited destination were included. Questions included demographic data (gender, age, nationality, education, profession), travel-related characteristics (destination country, duration of stay, influenza risk perception, previous travel health advice, travel purpose, travel costs) and general attitudes and practices towards influenza vaccination (vaccination coverage, reasons to be vaccinated, reasons to refuse vaccination, motivations to consider vaccination with options for multiple answers except for the vaccination coverage). In 2010, an additional question targeting the pandemic influenza A/H1N1 vaccination coverage was included. The questionnaires were checked for completeness. A written letter of exempt was received by the Ethical Commission of the Canton of Zurich.

Statistical analyses were conducted by using SPSS^® ^version 14.0 for Windows. Differences in proportions of demographics, travel-related data and attitudes and practices were compared using the Chi-square test. The significance level was set at p ≤ 0.05. For the multiple logistic regression analysis the surveys were analysed as well as pooled dataset and each survey, CTH-2009 and CTH-2010, separately. The seasonal influenza vaccination was used as outcome and all demographic, travel-related and attitude- and practices-related factors were evaluated as independent predictors. Odds Ratios (OR) were determined by stepwise backward elimination of variables with p > 0.150. For sensitivity analyses, each dataset of the CTH studies, 2009 and 2010, was analysed separately and additionally, predictors for pandemic influenza vaccination were determined by multiple logistic regression analyses.

## Results

### Study population

**Figure 1:**
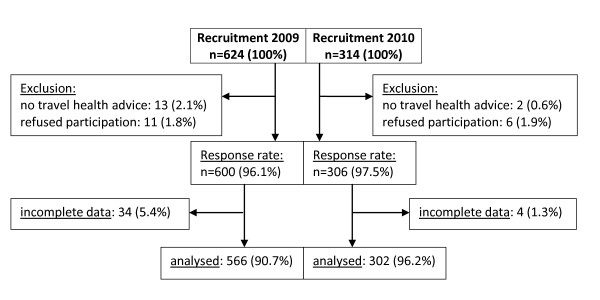
**Flow diagram of participants**.

From a total of 938 eligible individuals, 868 (92.5%) were included in the analysis (Figure 1). Overall, 479 (55.2%) were females and 389 (44.8%) males. The great majority of participants (503, 57.9%) were between 18 and 35 years old with a median age of 32 years (range 18 - 84 yrs). Only 46 (5.3%) responders were above 64 years of age. In general, participants were highly educated with 480 (55.3%) being university graduates. Overall, the characteristics of participants planning to travel to resource-limited destinations are presented in Table 1.

**Table 1:** Characteristics of participants planning to travel to resource-limited destinations

Characteristics		All (n = 868)	CTH 2009 (n = 566)	CTH 2010 (n = 302)	p-value
Gender:	female	479 (55.2%)	313 (55.3%)	166 (55%)	p = 0.925
	male	389 (44.8%)	253 (44.7%)	136 (45%)	

Age (yrs):	mean	37	37	37	p = 1.000
	median (range)	32 (18-84)	32 (18-81)	32 (18-84)	
Age-groups:	18 - 35 yrs	503 (57.9%)	324 (57.2%)	179 (59.3%)	p = 0.564
	36 - 50 yrs	204 (23.5%)	142 (25.1%)	62 (20.5%)	p = 0.131
	51 - 64 yrs	115 (13.2%)	74 (13.1%)	41 (13.6%)	p = 0.835
	> 64 yrs	46 (5.3%)	26 (4.6%)	20 (6.6%)	p = 0.204

Educational level:	prim./sec. school	20 (2.3%)	13 (2.3%)	7 (2.3%)	
	traineeship/A-level	368 (42.4%)	241 (42.6%)	127 (42.1%)	
	university	480 (55.3%)	312 (55.1%)	168 (55.6%)	p = 0.989

Travel continent:	Africa	243 (28%)	151 (26.7%)	92 (30.5%)	p = 0.237
	Latin America	273 (31.5%)	198 (35%)	75 (24.8%)	p = 0.002
	Asia	331 (38.1%)	208 (36.7%)	123 (40.7%)	p = 0.250
	SE Europe	6 (0.7%)	2 (0.4%)	4 (1.3%)	p = 0.744
	Oceania	15 (1.7%)	7 (1.2%)	8 (2.6%)	p = 0.128

Duration of stay (days):	1 - 7 days	29 (3.3%)	18 (3.2%)	11 (3.6%)	
	8 - 14 days	183 (21.1%)	112 (19.8%)	71 (23.5%)	
	15 - 28 days	342 (39.4%)	233 (41.2%)	109 (36.1%)	p = 0.432
	> 28 days	314 (36.2%)	203 (35.9%)	111 (36.8%)	

Perceived risk of influenza at destination:	high	72 (8.3%)	42 (7.4%)	30 (9.9%)	
	low	515 (59.3%)	338 (59.7%)	177 (58.6%)	p = 0.436
	no idea	281 (32.4%)	186 (32.9%)	95 (31.5%)	

Source of travel health information*:	internet	340 (39.2%)	211 (37.3%)	129 (42.7%)	p = 0.118
	family doctor	111 (12.8%)	74 (13.1%)	37 (12.3%)	p = 0.730
	family/friends	75 (8.6%)	50 (8.8%)	25 (8.3%)	p = 0.781

Purpose of the journey:	holidays	695 (80.1%)	448 (79.2%)	247 (81.8%)	p = 0.354
	VFR**	107 (12.3%)	73 (12.9%)	34 (11.3%)	p = 0.484
	business	92 (10.6%)	61 (10.8%)	31 (10.3%)	p = 0.815

Travel expenses (CHF):	0 - 4999	564 (65%)	378 (66.8%)	186 (61.6%)	
	5000 - 9999	197 (22.7%)	122 (21.6%)	75 (24.8%)	
	10000-15000	49 (5.6%)	35 (6.2%)	14 (4.6%)	p = 0.091
	> 15000	31 (3.6%)	15 (2.7%)	16 (5.3%)	

Seasonal vaccination coverage		119 (13.7%)	65 (11.5%)	54 (17.9%)	p = 0.009
Pandemic vaccination coverage		43 (14.2%)	na	43 (14.2%)	na

*prior to receiving travel health advice at the CTH (Centre for Travel Health)

VFR: people visiting friends or relatives, na: not available

p was determined with Chi-square test, statistical significance was set at p ≤ 0.05.

### Travel characteristics

The majority reported travel to South America (188, 21.7%), followed by South-east Asia (170, 19.6%) and South-central Asia (135, 15.6%). Most frequently countries planned to visit included India (125, 14.4%) and Thailand (87, 10%). Most travellers stayed at their destination between 15 days to four weeks (342, 39.4%). Only 72 (8.3%) travellers estimated their destination as at high risk for influenza infection, of these 17 (23.6%) were vaccinated against seasonal influenza. Only 111 (12.8%) responders were informed by their family physicians in advance, web-based information was consulted by 340 travellers (39.2%). The great majority, 695 (80.1%), included holiday travellers, 107 (12.3%) were visiting friends or relatives (VFR) and every tenth (92, 10.6%) was a business traveller.

### Vaccination coverage

A total of 119 (13.7%) participants were vaccinated against seasonal influenza, 43 (14.2%) against pandemic influenza in 2010 and 25 (8.3%) have received both influenza vaccinations. The great majority (630, 72.6%) has never received an influenza vaccination in their life. In the multiple logistic regression analysis, increasing age (OR = 1.03, 95% CI 1.01 - 1.04) and seasonal influenza vaccination in the previous winter seasons (OR = 12.91, 95% CI 8.09 - 20.58) were significant predictors for being vaccinated against seasonal influenza (Table 2). Business travellers (OR = 0.39, 95% CI 0.17 - 0.92) were significantly less often vaccinated than other traveller groups. The same independent factors were determined when analysing each survey, CTH-2009 and CTH-2010, separately. Having received the pandemic influenza vaccination was significantly associated with having received the seasonal influenza vaccination in winter 2009/2010 (OR = 10.86, 95% CI 5.11 - 23.09).

**Table 2:** Predictors of seasonal influenza vaccination among travellers to resource-limited destinations determined by multiple logistic regression analysis

Variable	Univariate OR (95% CI) N = 868	Multivariate OR (95% CI) N = 868	Final Model OR (95% CI) N = 868
Gender	1.05 (0.71 - 1.56)	1.10 (0.69 - 1.76)	1.11 (0.70 - 1.75)

Age (yrs)	1.05 (1.03 - 1.06)	1.03 (1.01 - 1.04)	1.03 (1.01 - 1.04)

Nationality	0.98 (0.92 - 1.04)		

Educational level	0.97 (0.68 - 1.38)		

Travel continent	0.95 (0.76 - 1.20)		

Destination by N vs. S hemisphere	1.49 (1.01 - 2.19)	1.47 (0.93 - 2.35)	

Duration of stay (d)	0.74 (0.59 - 0.93)	0.74 (0.56 - 0.99)	0.78 (0.59 - 1.03)

Perceived risk of influenza at destination	0.74 (0.54 - 1.03)	0.85 (0.52 - 1.26)	

Travel health advice by:			
family doctor	2.05 (1.25 - 3.36)	1.50 (0.78 - 2.89)	
travel clinic	1.25 (0.84 - 1.86)		
internet	0.73 (0.48 - 1.09)	0.79 (0.46 - 1.34)	
travel agency	0.57 (0.24 - 1.34)		
family or friends	0.85 (0.41 - 1.75)		
No advice	0.67 (0.41 - 1.09)	0.78 (0.42 - 1.46)	

Travel purpose:			
holidays	1.27 (0.76 - 2.12)		
business	0.57 (0.27 - 1.21)	0.40 (0.17 - 0.97)	0.39 (0.17 - 0.92)
VFR*	1.12 (0.64 - 1.99)		
education	0.58 (0.23 - 1.47)		
others	0.42 (0.10 - 1.80)		

Travel expenses	1.35 (1.08 - 1.69)	1.14 (0.84 - 1.56)	

Previous seasonal influenza vaccination	14.39 (9.18 - 22.58)	12.42 (7.71 - 20.01)	12.91 (8.09 - 20.58)

*people visiting friends or relatives

### KAP on seasonal influenza vaccination

Of all vaccinated participants, 44 (37%) declared to be vaccinated for business reasons and 25 (21%) due to age (Table 3). Only 10 (8.4%) responders reported to be vaccinated for their journey. Travellers remained mostly unvaccinated (326, 43.5%) because they felt not at risk, 144 (19.2%) have missed any recommendation by their family physician and a substantial proportion (172, 23%) did not see any relevance for being vaccinated. Often mentioned as "other reasons" for being unvaccinated (108, 14.3%) included items like: rare/never affected by influenza (43, 5.7%), vaccine is not effective enough (34, 4.5%) and bad experiences with vaccine/side-effects of vaccine (31, 4.1%). Most travellers would consider vaccination if they would feel in bad general health (408, 47%), followed by a recommendation of the family physician (327, 37.7%) and travel to regions with known high risk of influenza (305, 35.1%). Of note, 65 (7.5%) intended to refuse influenza vaccination at any time.

**Table 3:** Attitudes and practices regarding influenza vaccination among travellers to resource-limited destinations

Reasons for getting vaccinated (only vaccinated participants)	All (n = 119)	CTH 09 (n = 65)	CTH 10 (n = 54)	p-value
Because it is required for my job	44 (37%)	26 (40%)	18 (33.3%)	p = 0.453
My family doctor advised me to do it	27 (22.7%)	18 (27.7%)	9 (16.7%)	p = 0.153
Because of my age	25 (21%)	12 (18.5%)	13 (24.1%)	p = 0.454
Because it is free	8 (6.7%)	6 (9.2%)	2 (3.7%)	p = 0.231
Because of the arranged journey	10 (8.4%)	2 (3.1%)	8 (14.8%)	*p = 0.022*
Because of media attention on SARS and avian flu	0 (0%)	0 (0%)	na	na
Because of media attention on pandemic 2009	2 (3.7%)	na	2 (3.7%)	na

**Reasons for not getting vaccinated (unvaccinated participants)**	**All (n = 749)**	**CTH 09**	**CTH 10**	**p-value**
		**(n = 501)**	**(n = 248)**	

I made bad experiences with a previous flu vaccine	31 (4.1%)	23 (4.6%)	8 (3.2%)	p = 0.377
The vaccine is not effective enough	34 (4.5%)	30 (6.0%)	4 (1.6%)	*p = 0.007*
I did not get any recommendations	144 (19.2%)	114 (22.8%)	30 (12.1%)	*P < 0.001*
I am not at risk	326 (43.5%)	197 (39.3%)	129 (52%)	*p = 0.001*
It is not important	172 (23%)	115 (23%)	57 (23%)	p = 1.000

**Encouraging factors for vaccination next season**	**All (n = 868)**	**CTH 09(n = 566)**	**CTH 10(n = 302)**	**p-value**

If I would feel in bad general health	408 (47%)	260 (45.9%)	148 (49%)	p = 0.388
If my family doctor recommended it to me	327 (37.7%)	210 (37.1%)	117 (38.7%)	p = 0.635
Travel to regions with high risk of influenza	305 (35.1%)	203 (35.9%)	102 (33.8%)	p = 0.539
Because of age	215 (24.8%)	146 (25.8%)	69 (22.8%)	p = 0.338
Job requirement	233 (26.8%)	143 (25.3%)	90 (29.8%)	p = 0.151
Ongoing flu season	87 (10%)	59 (10.4%)	28 (9.3%)	p = 0.590
I would never be vaccinated	65 (7.5%)	40 (7.1%)	25 (8.3%)	p = 0.518

CTH: Centre for Travel Health

Multiple answers were possible. The p-value was determined with Chi-square test, statistical significance was set at p ≤ 0.05.

## Discussion

Travel as risk factor for an influenza infection is poorly established among international travellers when regarding the low vaccination coverage as well as the low self-perceived travel-associated risk estimates. Of note, previous influenza vaccinations facilitated receiving an influenza vaccination in the following year by about 13 times. Therefore, easy access to the influenza vaccine is important. High media coverage was not considered sufficient to increase the vaccination rate substantially as is indicated by the low increase of the vaccination coverage between the two surveys in 2009 and 2010 and also by the low pandemic influenza vaccination coverage of only 14.2%. Therefore, multiple efforts need to complement one another including information strategies provided by family physicians and travel medicine practitioners, but also job- and age-related activities need to be considered.

Our sample of travellers is comparable to other studies performed at our Centre for Travel' Health [7] with respect to the age distribution, educational level and travel duration. Inherent limitations include a selection bias: Frequently visited destinations such as the Middle East, North Africa and the Caribbean are underrepresented as travellers to those destinations generally do not consider a pre-travel health consultation as indicated [11] but destinations with higher risk for faecal-orally transmitted infectious diseases, such as TD or bacterial meningitis, are well represented, such as e.g. India and Sub-Saharan countries. Therefore, our sample may represent a best practice sample. The fact, that the high proportion of university graduates indicates a health literate population may result in an even overestimated risk perception as well as influenza vaccination coverage. All data collections relied on self-reported information. Hence, the results of the studies might be limited by a potential bias such as disclosure bias, although self-report of influenza vaccination status has been found to be reliable when checked against medical record documentation [13].

Most seasonal influenza activity occurs during November to April on the Northern hemisphere and vaccination is usually administered between October and November. Therefore, travellers visiting the opposite hemisphere have to be counselled accordingly and the seasonal influenza vaccine also for the Southern hemisphere has to be available as there is year-round influenza activity in tropical and subtropical areas.

## Conclusions

Risk perception and vaccination coverage regarding seasonal and pandemic influenza was very poor among European travellers to resource-limited destinations when compared to traditional at-risk groups. Age and previous influenza vaccination were the best predictors for considering seasonal influenza vaccination in our population. Communication strategies about influenza should be intensified and should include all health professionals, e.g. family physicians and travel medicine practitioners, but also business enterprises.

## Competing interests

The authors declare that they have no competing interests.

## Authors' contributions

TDS, MM and CH conceived and supervised the study. AP performed all data collection and data analysis and drafted the manuscript. MM participated in designing the study and the questionnaire and organised access to the data of the airport-study. All authors have read and approved the final manuscript.

## Pre-publication history

The pre-publication history for this paper can be accessed here:

http://www.biomedcentral.com/1471-2458/10/402/prepub
